# Possible Role of Chest Ultrasound in the Assessment of Costo-Phrenic Angle Lesions Prior to Medical Thoracoscopy: A Retrospective Pilot Case Series

**DOI:** 10.3390/diagnostics12112587

**Published:** 2022-10-25

**Authors:** Emanuele Giovanni Conte, Andrea Smargiassi, Filippo Lococo, Giampietro Marchetti, Riccardo Inchingolo

**Affiliations:** 1 Pulmonology Unit, “C.&G. Mazzoni” Hospital, 63100 Ascoli Piceno, Italy; 2 UOC Pneumologia, Dipartimento Scienze Mediche e Chirurgiche, Fondazione Policlinico Universitario A. Gemelli IRCCS, 00168 Rome, Italy; 3 Thoracic Surgery Unit, Dipartimento Scienze Mediche e Chirurgiche, Fondazione Policlinico Universitario A. Gemelli IRCCS, 00168 Rome, Italy; 4 Respiratory Medicine Department, ASST Spedali Civili, 25123 Brescia, Italy

**Keywords:** chest ultrasound, medical thoracoscopy, pleural effusion, pleural malignancy

## Abstract

Background: Pleural malignancy (PM) and malignant pleural effusion (MPE) represent an increasing burden of diseases. Costo-phrenic angle (CPA) could be involved by malignant small nodularities or thickenings in the case of MPE. The aim of this study was to evaluate whether lung ultrasound (LUS), performed prior to medical thoracoscopy (MT), could detect pleural abnormalities in CPA not easily detectable by chest computed tomography scan (CCT). Methods: Patients suspected for PM and MPE were retrospectively recruited. Patients underwent both LUS examination with a linear array and CCT prior to diagnostic medical thoracoscopy. LUS pathological findings in CPA were compared with pathological findings detected by CCT. Findings were confirmed by subsequent MT, the gold standard for PMs. Results: Twenty-eight patients were recruited. LUS detected 23 cases of pleural abnormalities in CPA. CCT was detected 12 pleural abnormalities. Inter-rater agreement between the two techniques was minimal (Cohen’s Kappa: 0.28). MT detected PMs in CPA in 22 patients. LUS had a sensitivity of 100% and specificity of 83%. CCT had a sensitivity of 54% and specificity of 100%. A better sensitivity for CCT was reached analysing only all abnormalities > 5 mm (64.3%). Conclusions: LUS examination, in the case of PMs, could change and speed up diagnostic workup.

## 1. Introduction

Pleural effusion (PE) is a common medical problem in patients hospitalized in pneumology or internal medicine departments, and aetiology varies according to geographical area, healthcare setting, patient age and other factors. An important category of PE is malignant pleural effusion (MPE). Epidemiological data suggest that MPE is one of the top three causes of pleural effusion, along with heart failure and para-pneumonic effusions [1]. The majority of MPE is caused by metastatic disease, most commonly lung cancer in men and breast cancer in women. These two cancers account for 50–65% of all MPE [1]. Mesothelioma is the most common type of primary pleural tumour and is associated with MPE in more than 90% of cases [2].

Nowadays, the gold standard in pleural disease assessment is medical thoracoscopy [2,3]. Currently, the diagnostic yield in patients with malignant pleural disease is reaching 94–97% [4,5].

Chest CT scan is considered as the most important radiological technique in evaluating pleural surface. Pleural Diseases BTS guidelines recommend performing this exam in the case of exudative pleural effusion without diagnosis after thoracentesis [6]. Anyway, several studies reported contrasting data on CT scan sensitivity and specificity [7,8,9]. The work published by Tsim and coworkers reported CT sensitivity of 58% and specificity of 80% in detecting pleural malignancies, concluding that radiological examination is not sufficient to exclude or confirm presence of primary or secondary pleural malignancies [10], thus requiring invasive procedures.

Another important technique in assessing pleural disease is chest ultrasonography. Nowadays, respiratory physicians routinely use thoracic ultrasound, mainly for the guidance of pleural interventions to minimise complications [11]. International guidelines strongly recommend the utility of this technique [12]. Chest US can discriminate features highly specific for malignancy and may therefore help to expedite correct investigations in those with these high-risk features [12,13]. In the case of pleural effusions, Chest US is able to assess, with high sensibility, pleural abnormalities especially at the costo-phrenic angle (CPA) [14].

It is crucial to study CPA. This region is the most caudal area of the thorax, it is delimitated by parietal, diaphragmatic and visceral pleura, each one characterized by different lymphatic drainage. It is rich of lymphatic stomata [15] being pulled open by inspiratory movements of lung and thoracic cage. Francisco Rodriguez-Panadero and colleagues detected that the majority of the pleural malignancies are caused by tumour emboli to the visceral pleura with subsequent secondary seeding to the parietal pleura [16].

It has been demonstrated that malignant seeding can be influenced by gravitational effects for intra-abdominal distribution [17]. Similarly, it has been described an increased prevalence of pleural abnormalities in the lower posterior area of thorax [18]. Pleural seeding and stasis of pleural fluid in this region lead us to focus our research to this anatomical area to find neoplastic lesions.

Moreover, chest ultrasound has been reported to have an excellent diagnostic accuracy for small pleural lesions, guiding percutaneous pleural needle biopsy. Percutaneous ultrasound guided pleural biopsy has high diagnostic yield and low complication rate [19]. Park J and colleagues [20] reported that ultrasound guided pleural biopsy is highly likely diagnostic for small pleural lesions with nodular morphology on either CT or US or with a pleural thickness of 4.5 mm or greater.

Aim of this study is to provide a picture of real life in a Pleural Unit, evaluating whether lung ultrasound (US), performed prior to medical thoracoscopy, could detect pleural abnormalities in CPA sometimes not easily detectable by chest computed tomography (CT) scan, previously performed and brought for viewing in the outpatient visit.

## 2. Materials and Methods

### 2.1. Study Population

In this retrospective case series, we included patients referred to Pleural Unit (Spedali Civili, Brescia, Italy) during a 38-month period who underwent Medical Thoracoscopy for suspected PMs, pleural effusions, or pleural abnormalities, already subject to chest ultrasonography with at least one chest CT scan. Patients were selected through a pleural disease database. The inclusion criteria were: (1) age ≥ 18 years; (2) suspect of pleural malignancies, pleural effusions or pleural abnormalities less than 10 mm; (3) chest ultrasound evaluation of costo-phrenic angle prior to medical thoracoscopy; (4) chest CT scan evaluation in the 30 days prior to medical thoracoscopy. Written informed consent was obtained from all patients retrospectively involved. This study was approved by the ethics committee of the Spedali Civili of Brescia (CE133/2017).

### 2.2. Chest Ultrasonography

Ultrasonographic assessment was performed using MyLabTM 30 CV machine (Esaote, Genova, Italy) equipped with convex (2–5 MHz) and linear (7–13 MHz) probes. All ultrasonographic evaluations were performed by a pneumologist (GM) with a consolidated expertise in lung ultrasonography.

Each patient was asked initially to stay seated for dorsal sonographic scans, then to lie in a supine position for anterior and lateral scans and finally in the lateral thoracoscopic decubitus position. A bilateral ultrasonographic evaluation was performed.

The convex probe was used firstly to look for pleural effusions, large lung consolidations and curtain signs. Secondly, the linear probe (7.5 MHz) was used to detect sliding sign, adherences, pleural thickenings, and small pleural abnormalities. CPA was constantly evaluated searching for small pleural thickenings and subcentrimetric nodules. Images and videos of costo-phrenic angle pleural lesions were acquired and stored.

Videos of the sonographic assessment of CPA were recorded and stored. A subsequent evaluation by 3 pneumologists (EGC, AS and RI) with high expertise in lung ultrasonography was performed for this retrospective study.

Pleural findings were classified, according to previous studies [21,22], in pleural thickenings and pleural nodules. Each lesion was measured and categorized.

### 2.3. Chest CT Scan

We reviewed all chest CT scans reports and images performed on enrolled patients. Chest CT scans have already been performed previously and brought for viewing in the visit at the Pleural Unit. Radiological examinations were not performed in the same centre and different CT scanner, parameters and protocols were reported. Contrast enhancement evaluation was not undertaken in all examinations.

We searched for description of pleural lesions in the costo-phrenic angle. The presence or absence of lesions was reported.

### 2.4. Medical Thoracoscopy (MT)

All MT procedures were performed in the Pleural Unit of ASST Spedali Civili (Brescia, Italy) by pulmonologists in a dedicated Endoscopy Room. Anaesthetists assisted patients during procedures providing conscious sedation.

Patients were placed in the lateral decubitus position with the ipsilateral arm abducted to maximize access to the hemi thorax. Chest ultrasonography was also performed immediately before the MT with the patient already in the lateral decubitus in order to detect sliding sign and the best entry site [23,24].

Local anaesthesia was induced with mepivacaine (200 mg) and after making a small skin incision, blunt dissection was performed with a curved blunt-point scissors in the chest wall until penetration of parietal pleura. Subsequently, a blunt-point trocar was carefully introduced through the chest wall, reaching pleural cavity. After aspiration of pleural fluid, a rigid 7-mm thoracoscopy set (Karl Storz GmbH & Co., Tuttlingen, Germany) was introduced in the pleural cavity. A complete assessment of pleural cavity was performed, and images and videos were acquired and stored in a local hard-disk. At least eight pleural biopsy specimens for each patient were then collected. A detailed report of the procedure, with description of macroscopic features of lesions, was stored in the Pleural Unit database.

### 2.5. Statistical Analysis

A descriptive analysis was accomplished by computing mean values and standard deviations. Kappa statistic was used to assess agreement between ultrasound and computed tomography technique. Linear weighted kappa was calculated for the ordered categories [25]. Finally, we calculated sensitivity, specificity, and receiver operating characteristic (ROC) curve by comparing the results that were obtained from chest US and chest CT scan respect to gold standard MT. Data analysis was performed using MedCalc Statistical Software version 17.6 (MedCalc Software bvba, Ostend, Belgium; http://www.medcalc.org (accessed on 8 December 2017)).

## 3. Results

The study population (Table 1) consisted of 28 patients, 21 males and 7 females, with a mean age of 64 ± 5 years (range 19–81). Malignancies were detected in final diagnosis in 22 cases (78.5%). Benign or infectious diseases were found in six cases. Pleural effusion was present in 25 cases (89% of subjects) and it was most frequently right sided (15 cases).

**Figure 1:**
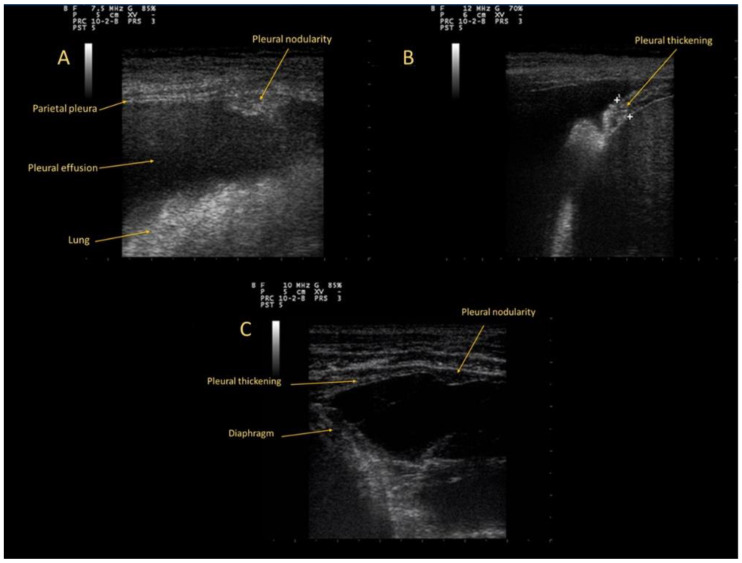
(**A**): Pleural nodularity; (**B**): Pleural thickening; (**C**): pattern of nodules and thickenings.

All patients underwent chest US examination. Pleural abnormalities in CPA were detected in 23 patients (82%). These abnormalities (Figure 1) were classified in: pleural thickenings (12 cases), nodularities (seven cases) and a combination pattern of nodules and thickenings (four cases). Each pattern was divided into two categories based on dimension: up to 5 mm, and ranging from 5 to 10 mm. (Table 2).

Chest CT scan was performed with and without contrast enhancement in 28.6% (8) and 71.4% (20) of patients, respectively. Pleural abnormalities in CPA were detected by chest CT scan in 12 patients (43%).

### 3.1. Chest US and Chest CT Scan Inter-Rater Agreement

Inter-rater agreement between chest US and chest CT scan findings was evaluated (Table 3).

Both techniques detected pleural abnormality in CPA in 12 patients. Agreement for the absence of pathological findings was reported in 5 patients.

In 11 cases, only US evaluation detected pleural abnormalities. No cases were reported for which CPA abnormalities, detected by chest CT scan, were non detectable with chest US evaluation.

Inter-rater agreement between the two techniques was assessed by linear weighted kappa values. Cohen’s Kappa was 0.28 (95% CI 0.050–0.51). This result described a minimal concordance between chest US and chest CT scan.

### 3.2. Comparison with Gold-Standard MT

Medical thoracoscopy detected pleural abnormalities in the CPA in 22 patients (79%). From macroscopic point of view, 10 patients had nodularities, nine pleural thickenings, and three patients both abnormalities.

Concerning pleural biopsies, a final diagnosis of pleural malignancy was achieved in 22 patients (79%). Ten patients suffered from mesothelioma (35%), five lung cancer (18%), two pleural metastasis of breast cancer (7%), and five other malignancies (ovarian; bone; kidney; solitary fibrous tumor; myoepithelial (Table 1).

Six patients (21%) had a final non-malignant diagnosis: two cases of pleural tuberculosis (7%) and four cases of unspecified pleural inflammation (14%).

**Figure 2:**
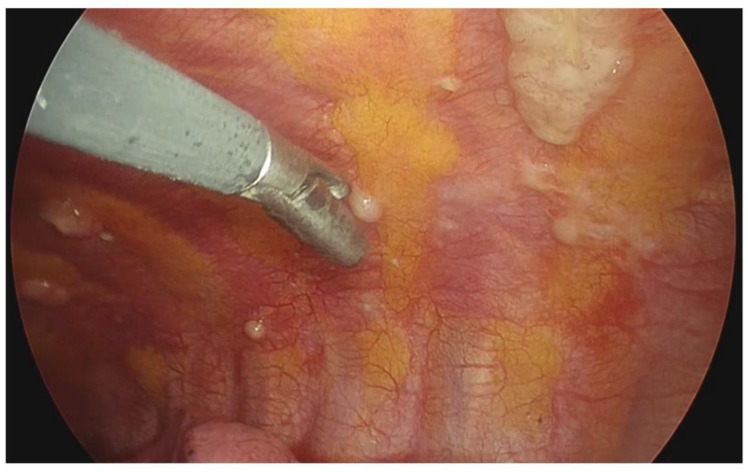
Thoracoscopic view: small pleural lesions on the surface of parietal pleura. After biopsy, pathological examination confirmed a pleural carcinosis from NSCLC.

Comparing ultrasound findings to medical thoracoscopy (Figure 2), MT confirmed the presence of pleural abnormalities in 22 of 23 cases detected by chest US. Only one false-positive was reported, resulting to be diaphragmatic pillars at MT examination. In the remaining five patients, no abnormalities were found by MT in agreement with chest US (Table 4).

Comparing radiological findings to MT, CT scan correctly detected presence of abnormalities in 12 cases, absence of abnormalities in six cases, but in 10 cases it was falsely negative (Table 4).

A comparison of sensitivity, specificity for both chest US and chest CT scan versus MT findings in CPA is reported in Table 5.

When compared to MT, chest CT scan had a sensitivity of 54% (95% CI 32.2% to 75.6%) and specificity of 100% (95% CI 54% to 100%).

Similarly, chest US had instead a sensitivity of 100% (95% CI 84.5% to 100%) and specificity of 83% (95% CI 36% to 99.6%).

**Figure 3:**
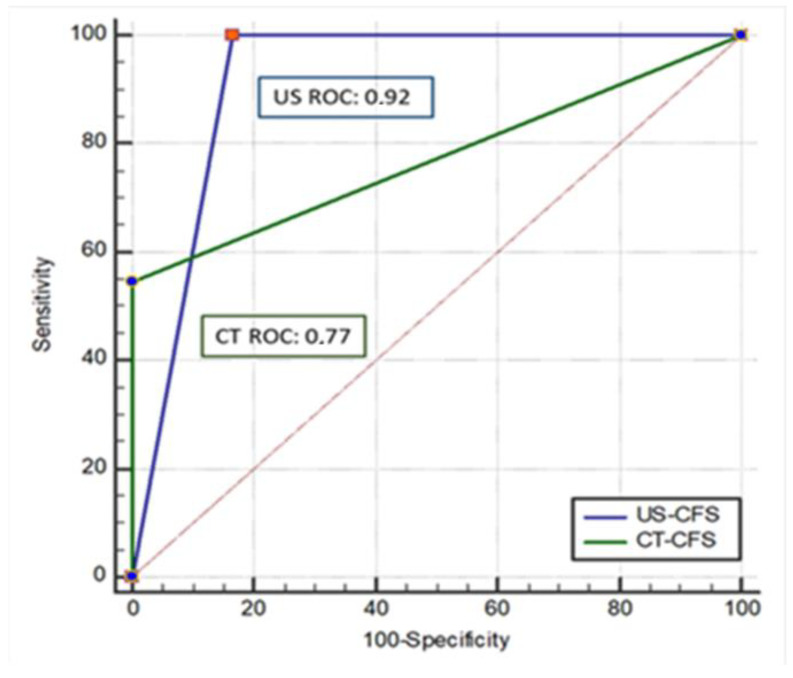
ROC curves for chest US and chest CT scan.

We calculated the ROC curve for each of the two techniques (Figure 3), showing an area under the curve (AUC) for chest US of 0.92 and for chest CT scan of 0.77 (*p* = 0.148).

In order to explain differences between chest US and chest CT scan in terms of sensitivities versus gold standard, we compared chest CT to medical thoracoscopy in a subgroup analysis of patients according to dimension of abnormalities detected by Chest US (Table 6).

When all patients with pleural abnormalities <5 mm (8 patients) were included in the analysis, chest CT scan demonstrated 37.5% of sensitivity, instead in patients with pleural abnormalities >5 mm (14 patients), CT scan demonstrated 64.3% of sensitivity.

Finally, considering the two subgroups separately, when the first CT scan of the chest was performed with (eight cases) and without (20 cases) iodine contrast of mean, it was possible to compute sensitivities and specificities differentiating cases.

Although low number of cases are reported, for the eight cases with contrast enhanced CT scan of the chest, sensitivity versus gold standard MT, was 87% for CT scan and 100% for chest US, respectively. It is not possible to report specificities because all eight cases were positive for CPA at MT (Table 7).

On the other hand, for the 20 cases without contrast enhanced CT scan of the chest, sensitivity versus gold standard MT, was 36% for CT scan and 100% for chest US. In these cases, specificities versus gold standard MT were 100% for CT scan and 83% for chest US, respectively (Table 8).

## 4. Discussion

This case series showed how chest US is able to help physicians for the assessment of CPA in case of suspected pleural malignancies. It is able to detect pleural abnormalities in CPA not easily detectable by first chest CT scan, especially in the case of absence of iodine mean of contrast. These abnormalities have been confirmed by MT which can be considered the referral technique.

Studying CPA, chest US detected all cases (22 out of 22) of pleural abnormalities identified by medical thoracoscopy (Table 4). Only one false-positive case has been reported: an apparent nodular lesion on the diaphragmatic surface of the CPA, resulting in an abnormal diaphragmatic pillar at thoracoscopic evaluation. In five cases chest US reported the absence of alterations, confirmed by MT. Chest US, when compared to MT, showed an overall sensitivity of 100% and a specificity of 83%.

As to chest CT scan, it detected only 12 cases out 22 reported by MT. However, no false-positive case has been reported. These results are similar to other described in current medical literature [8,9].

Chest CT scan in our series demonstrated a high specificity in detecting pleural abnormalities in the CPA (100%), but a low level of sensitivity (54%).

ROC analysis for chest US and CT scan showed higher accuracy for the ultrasound evaluation of CPA if compared to chest CT, although the difference between AUCs was not significant. Comparison between chest US and chest CT scan resulted in a minimal concordance, assessed by the Cohen’s Kappa. Discordance was detected in 11 cases. In all cases, chest US was positive for pleural abnormalities in CPA and chest CT scan negative. In 10 out of these 11 cases, MT actually identified abnormalities. One case confirmed the absence of pathology according to chest CT scan. Therefore, radiological examination was unable to detect 10 cases, correctly identified by chest US.

The majority of these cases were represented by small pleural abnormalities in CPA (usually less than 5 mm). Among these cases, we reported pleural malignancies, but also one patient with a histological diagnosis of unspecified pleural inflammation and two patients with pleural tuberculosis.

Based on these findings, we compared chest CT scan to medical thoracoscopy in a subgroup analysis of patients according to dimension of abnormalities detected by chest US. We found that CT scan detected with lower sensitivity abnormalities <5 mm (37.5%). A better sensitivity was reached for all abnormalities >5 mm (64.3%).

Our results could be explained by the different spatial resolution of US examination [23] with a high frequency probe compared to chest CT scan performed without high resolution protocol, not required in the study of suspected pleural malignant effusion or pleural malignancies [6].

Our study has several limitations. The first one is the retrospective model of our study. We could include in our work only patients who underwent a lung ultrasonography and a chest CT scan prior medical thoracoscopy and we excluded all patients whose images and videos were not recorded and all patients who performed a chest CT scan, with or without iodine contrast mean, after the procedure. Moreover, chest CT scan examinations were not performed in the same centre, with same protocols and all with contrast enhancement phase. Most of the exams were performed without contrast enhancement because of patients either with renal failure or with known adverse reactions to iodine contrast mean or because the first chest CT scan has been usually performed without contrast enhancement. Another limitation is the small population of our study and the higher proportion of patient affected by mesothelioma compared to other malignant diseases, above all lung cancer. This can be due to the high incidence of mesothelioma in the part of Italy that refers patients to the Pleural Unit of ASST Spedali Civili, Brescia [26].

Despite these limitations, our original observation suggests that the absence of pleural abnormalities detected by first chest CT scan is not sufficient to exclude pleural involvements especially in case of malignancy.

Chest US could improve detection of even millimetric pleural abnormalities localized in the costo-phrenic angle, not detected by chest CT scan, with high sensitivity and specificity when compared with gold standard medical thoracoscopy.

Even if diagnostic performance of chest CT scan is not sufficient to exclude or confirm small pleural abnormalities, it is crucial to assess mediastinal and diaphragmatic pleural surface, pleura behind ribs or shoulder blades, lung fissures, lung parenchyma, central nodules, or peripheral lung abnormalities not reaching pleural surface.

The aim of this work, presenting a picture of real life in pleural unit, was not to suggest chest US in substitution of chest CT scan, but to provide pulmonologists with a useful tool to assess pleural surface [27] and chest wall [28], in addition to ionizing radiations [29,30,31], in order to indicate and guide diagnostic MT.

Finally, this retrospective case series represents the first step towards a prospective study, enrolling patients with a standardized protocol, focusing on the role of chest US in the assessment of costo-phrenic angle prior to MT with the final goal to make this technique common in clinical practice.

## 5. Conclusions

According to our results, US showed a good sensitivity in the detection of pleural abnormalities localized in the costo-phrenic angle. An accurate ultrasound examination of CPA in patients affected by pleural effusion or suspected malignant pleural effusion could assess even millimetric pathological lesions, sometimes not easily detectable by chest CT scan.

**Figure 4:**
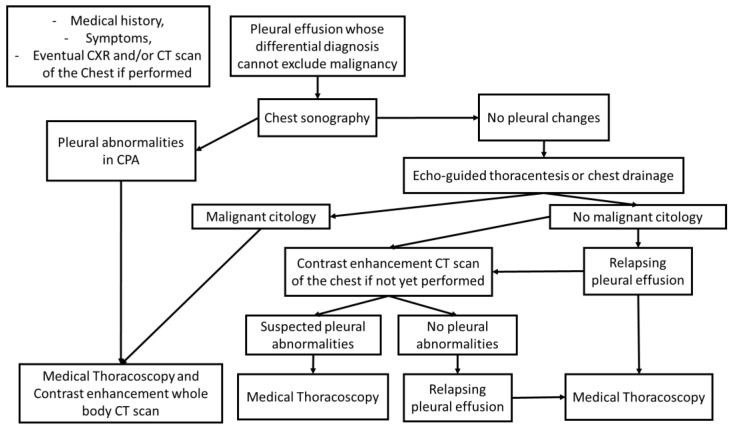
Proposal of diagnostic algorithm.

US examination, in the presence of a suspected pleural pathology, could change and speed up diagnostic workup (Figure 4) [13,14,32,33,34,35], aiding malignancy characterization and therapeutic care.

## Figures and Tables

**Table 1:** Patients’ characteristics.

Patient	Age	Sex	Effusion Side	Final Diagnosis
Patient 1	81	M	Right	Unspecified pleural inflammation
Patient 2	76	F	Right	Epithelioid mesothelioma
Patient 3	66	M	No pleural effusion	Lung Adenocarcinoma
Patient 4	66	M	Right	Unspecified pleural inflammation
Patient 5	75	M	Right	Epithelioid mesothelioma
Patient 6	76	F	Right	Breast Cancer
Patient 7	60	M	Right	Epithelioid mesothelioma
Patient 8	21	M	Right	Tuberculosis
Patient 9	78	M	Left	Biphasic mesothelioma
Patient 10	72	M	Right	Lung Adenocarcinoma
Patient 11	56	M	Left	Other malignancy
Patient 12	63	F	No pleural effusion	Epithelioid mesothelioma
Patient 13	68	M	Right	Lung Squamous cell carcinoma
Patient 14	68	F	Right	Epithelioid mesothelioma
Patient 15	75	M	Left	Epithelioid mesothelioma
Patient 16	49	M	Left	Tuberculosis
Patient 17	73	F	No pleural effusion	Epithelioid mesothelioma
Patient 18	64	M	Left	Epithelioid mesothelioma
Patient 19	48	M	Right	Lung Adenocarcinoma
Patient 20	49	F	Left	Breast Cancer
Patient 21	62	M	Right	Other malignancy
Patient 22	19	M	Left	Other malignancy
Patient 23	79	M	Right	Unspecified pleural inflammation
Patient 24	61	M	Left	Other malignancy
Patient 25	81	F	Right	Other malignancy
Patient 26	62	M	Left	Unspecified pleural inflammation
Patient 27	77	M	Left	Lung Adenocarcinoma
Patient 28	65	M	Right	Epithelioid mesothelioma
**Characteristic**				**Values**
Patients, n				28
Male/Female				21/7
Mean age (range), years				64 ± 5 (19–81)
***Effusion side (Ultrasound)***				
Left				10
Right				15
Absent				3
***Final diagnosis***				
*Benign*				*6*
Unspecified pleural inflammation				4
Tuberculosis				2
*Malignant*				*22*
Mesothelioma				10
Epithelioid mesothelioma				9
Biphasic mesothelioma				1
Lung cancer				5
Adenocarcinoma				4
Squamous cell carcinoma				1
Breast cancer				2
Other malignancies				5

**Table 2:** Chest US findings in CPA.

CPA US Findings	Values
*No abnormalities*	5
*Nodularities 5–10 mm*	7
*Pleural thickening*	12
<5 mm	5
5–10 mm	7
*Nodularities and pleural thickening*	4
<5 mm	3
5–10 mm	1

**Table 3:** Comparison of chest US and chest CT scan findings in CPA.

	*Chest Ultrasound*		
***Chest CT scan***	**Positive**	**Negative**	
**Positive**	12	0	12 (43%)
**Negative**	11	5	16 (57%)
	23 (82%)	5 (18%)	28

**Table 4:** Chest US and CT findings compared with MT gold standard.

	Disease Present (MT)	Disease Absent (MT)
**Chest US positive**	22	1
**Chest US negative**	0	5
**Chest CT positive**	12	0
**Chest CT negative**	10	6

**Table 5:** Sensitivity, Specificity, AUC-ROC analysis of Chest US and Chest CT scan vs. gold standard MT.

Diagnostic Test	Sensitivity	Specificity	AUC
**Chest ultrasound**	100%	83%	0.92
**Chest CT**	54%	100%	0.77

**Table 6:** Comparison between Chest CT scan vs. gold standard MT according to subgroup analysis of Ultrasonographic findings.

**All abnormalities < 5 mm**	8	37.5%	100%
**All abnormalities > 5 mm**	14	64.3%	100%

**Table 7:** Subgroup of 8 cases with contrast enhanced (c.e.) CT scan of the chest. Chest US and CT scan compared with MT gold standard for pleural abnormalities in CPA.

	Disease Present (MT)	Disease Absent (MT)
**Chest US positive**	8	0
**Chest US negative**	0	0
**c.e. CT scan positive**	7	0
**c.e. CT scan negative**	1	0
**Diagnostic test**	**Sensitivity**	**Specificity**
**Chest ultrasound**	100%	N.A.
**c.e. CT scan**	87%	N.A.

**Table 8:** Subgroup of 20 cases without contrast enhanced (c.e.) CT scan of the chest. Chest US and CT scan compared with MT gold standard for pleural abnormalities in CPA.

	Disease Present (MT)	Disease Absent (MT)
**Chest US positive**	14	1
**Chest US negative**	0	5
**CT scan positive**	5	0
**CT scan negative**	9	6
**Diagnostic test**	**Sensitivity**	**Specificity**
**Chest ultrasound**	100%	83%
**CT scan**	36%	100%

## Data Availability

The data presented in this study are available on request at emanuele.conte_86@hotmail.it.
